# Long Term Disease Control of Brain Metastases From Chemotherapy‐Resistant Endometrial Cancer With Lenvatinib and Pembrolizumab: A Case Report

**DOI:** 10.1111/jog.70102

**Published:** 2025-10-14

**Authors:** Ayaka Fujioka, Akimasa Takahashi, Tsukuru Amano, Yuji Tanaka, Yutaka Yoneoka, Shunichiro Tsuji

**Affiliations:** ^1^ Department of Obstetrics and Gynecology Shiga University of Medical Sciences Otsu Japan

**Keywords:** brain metastases, endometrial cancer, lenvatinib, pembrolizumab, targeted therapy

## Abstract

Brain metastases from endometrial carcinoma are extremely rare and associated with poor prognosis. We present a 43‐year‐old woman with mismatch repair deficient endometrial cancer who developed multiple brain metastases refractory to cytotoxic chemotherapy and without extracranial involvement. After stereotactic radiotherapy, combination therapy with lenvatinib and pembrolizumab resulted in sustained partial remission for 16 months, with no evidence of brain hemorrhage. This case demonstrates that lenvatinib plus pembrolizumab may offer an effective and safe therapeutic option for brain metastases from endometrial cancer, even in patients resistant to conventional therapy. Remarkably, this outcome challenges the traditionally poor prognosis of such cases and underscores the potential for novel targeted and immunotherapeutic strategies to redefine the standard of care for this rare and devastating complication.

## 1 Introduction

The number of new cases of endometrial cancer is increasing worldwide, with recurrence and mortality rates also increasing year on year [1]. In general, 70% of endometrial cancer cases are stage I cases, and the recurrence rate after appropriate treatment is not very high at 10.6% [2]. Common sites of recurrence in endometrial cancer are pelvic to para‐aortic lymph nodes, liver, lungs, and bones, with brain metastases being rare. The incidence of brain metastases in endometrial cancer is estimated to be 0.3%–1.16%, with survival from diagnosis of recurrence ranging from 3.5–6.5 months. With regard to the treatment of brain metastases from endometrial cancer, surgical resection and radiotherapy significantly prolong survival in cases with a single brain lesion and no extracranial involvement [3].

Recently, the study 309/KEYNOTE775 trial demonstrated the efficacy and safety of the combination of lenvatinib and pembrolizumab in patients with advanced endometrial cancer. There are limited data evaluating the therapeutic efficacy of multi‐targeted receptor tyrosine kinase inhibitors (TKIs) and immune checkpoint inhibitors in brain metastases from endometrial cancer. Lenvatinib, a novel molecularly targeted agent, is a multi‐targeted receptor tyrosine kinase inhibitor (TKI) approved by the Japanese regulatory authorities for the treatment of recurrent endometrial cancer. TKIs include the vascular endothelial growth factor (VEGF) receptor, fibroblast growth factor (FGF) receptor, and platelet‐derived growth factor (PDGF) receptor. Although TKIs are associated with an increased risk of bleeding into brain metastases, we chose this treatment for a patient with a metastatic brain tumor from uterine cancer who had not responded to initial treatment.

We report on the rare clinical course of this patient whose treatment with lenvatinib and pembrolizumab for multiple brain metastases of cytotoxic drug‐resistant endometrial cancer has inhibited tumor growth and resulted in long‐term survival without the side effect of brain hemorrhage.

## 2 Case Report

**FIGURE 1:**
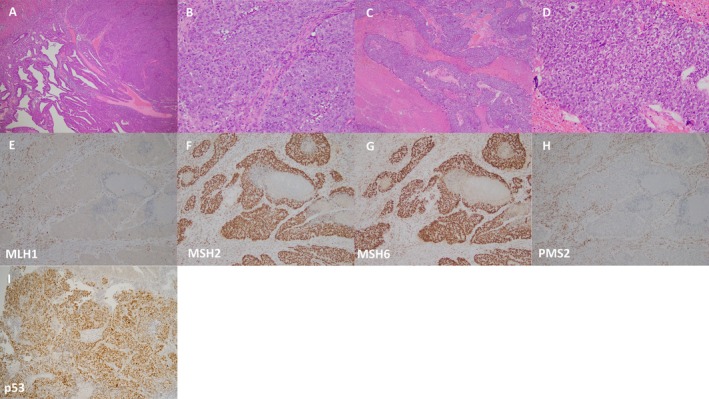
Hematoxylin Eosin staining and mismatch repair immunostaining of endometrial carcinoma. (A) Microscopic image of endometrioid carcinoma of the uterus at a magnification of 40×. (B) Microscopic image of endometrioid carcinoma of the uterus at a magnification of 200×. (C) Metastatic tumor cells from endometrial cancer of brain at a magnification of 40×. (D) Metastatic tumor cells from endometrial cancer of brain at a magnification of 200× (E) MLH1 demonstrate similar patterns of protein loss. (F) Staining was confirmed for MSH2 and MSH6. (G) Staining was confirmed for MSH6. (H) PMS2 demonstrates similar patterns of protein loss. (F, G) Staining was confirmed for MSH2 and MSH6. (I) Staining was confirmed for p53. All original images are captured at a magnification of 200×.

**FIGURE 2:**
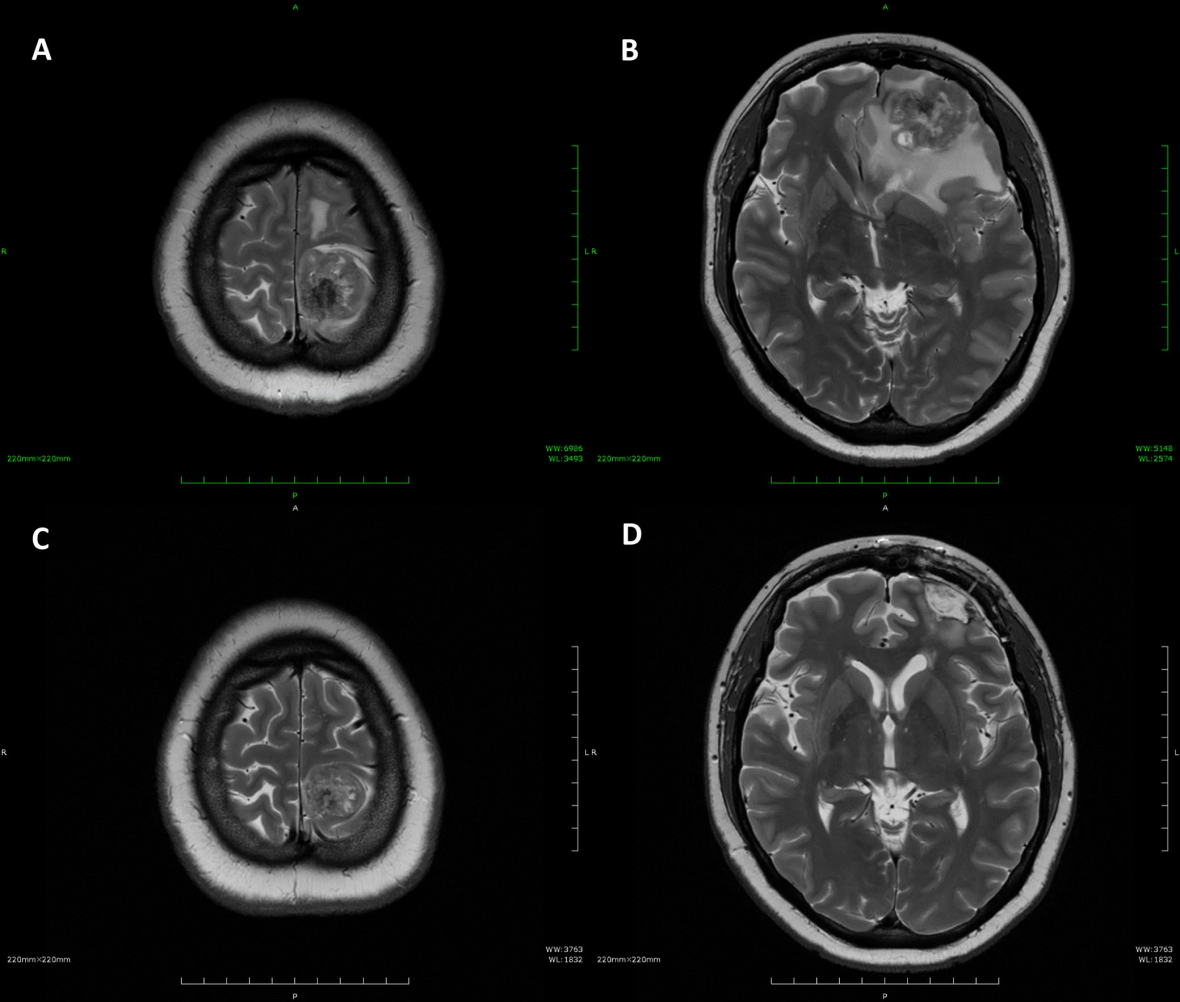
Magnetic resonance imaging of the brain. (A) T2‐weighted images revealed a substantial mass in the parietal lobe. (B) T2‐weighted images revealed a mass with midline shift in the frontal lobe. (C) T2‐weighted images showed shrinkage of the parietal lobe mass after irradiation and lenvatinib plus pembrolizumab treatment. (D) T2‐weighted images showed shrinkage of the frontal lobe mass after surgery, irradiation and lenvatinib plus pembrolizumab treatment.

A 43‐year‐old (2 gravida and 2 para) presented to the clinic with the main complaint of irregular bleeding. She was referred to our clinic with a diagnosis of endometrial cancer. CT and MRI examinations performed at our clinic showed no distant metastases, but there was seeding to the omentum and para‐aortic lymph node metastasis up to the renal vein. Total hysterectomy, bilateral adnexectomy, pelvic to para‐aortic lymph node dissection, and partial para‐aortic lymphadenectomy were performed, and complete resection was obtained grossly. Postoperative histopathology confirmed endometrioid adenocarcinoma, Grade 3, with mismatch repair defect (dMMR) (Figure 1). Additionally, p53 immunohistochemical staining showed diffuse strong positivity. Seeding into the large omentum was also observed, leading to a diagnosis of pT3aN2M1, stage IVB (FIGO2008). Post‐operative adjuvant chemotherapy with adriamycin 60 mg/m2 and cisplatin 50 mg/m2 was administered every 3 weeks, and at the fourth course visit, the patient complained of right lower limb weakness. A brain CT and MRI scan showed two mass shadows in the cerebrum. Brain tumor resection was performed, and a diagnosis of metastasis of uterine cancer was made. The residual lesions were irradiated with 30 Gy as Volumetric Modulated Arc Therapy (VMAT). The patient was then treated with lenvatinib 20 mg + pembrolizumab 200 mg, and by the third course, the numbness in the lower limbs had improved. After nine courses, MRI scans showed no tumor growth, and the patient was followed up (Figure 2). The patient developed grade 2 hypothyroidism and grade 2 erythema multiforme by the third course; however, these adverse events were adequately controlled by medication, allowing LP therapy to be continued without dose reduction. After the ninth course, the patient developed grade 3 enteritis, which necessitated discontinuation of both agents and initiation of steroid pulse therapy. Side effects of enteritis Grade 3, hypothyroidism Grade 2, and erythema multiforme Grade 2 were observed but were controlled by medication. Sixteen months have passed since the start of recurrence treatment, but partial remission has been maintained. Written informed consent for the publication of the patient's clinical data was obtained from the patient. The authors would like to thank the patient for providing consent to publish this case.

## 3 Discussion

Patients with metastatic brain tumors lacking lung or liver metastases who emerged during postoperative platinum‐based anticancer treatment for endometrial cancer were successfully treated with lenvatinib plus pembrolizumab to control disease progression. This is an extremely rare case report of successful lenvatinib plus pembrolizumab treatment for brain metastases from endometrial cancer.

Brain metastases in endometrial cancer have no effective treatment strategy and their prognosis is very poor. Brain metastases from endometrial cancer without lung or liver metastases are very rare [4]. The main route of metastasis in endometrial cancer is lymph node metastasis, with hematogenous metastases of lung, liver, bone, and brain metastases being rare [4]. The incidence of brain metastases in particular has been reported to be 0.2%–1.2% [5]. An autopsy study also reported an incidence of 1.1% [6]. Among single organ metastases, lung metastasis, liver metastasis, bone metastasis, and brain metastasis are the rarest metastases, at 0.06%, and the median OS is 5 months, the shortest among these four hematological metastases [4]. In other reports, once a brain metastasis is diagnosed, the average life expectancy is approximately 5 months, whether it is the first occurrence or a recurrence [3, 7]. Recently, a case was reported in which a patient with pMMR endometrial cancer and brain metastasis was treated with whole‐brain radiation therapy (WBRT) followed by lenvatinib plus pembrolizumab (LP) therapy, resulting in disease control for 15 months. However, the patient ultimately developed lung metastasis and died of primary disease at 31 months [8]. In our case, we performed stereotactic radiation therapy for the metastatic brain tumor, followed by LP therapy. In cases of symptomatic brain metastases such as the present case, it is important, as an onco‐emergency, to first control intracranial pressure with surgical resection and/or radiotherapy. When surgical intervention is performed, initiation of lenvatinib should be delayed for 4 weeks; therefore, radiotherapy during this waiting period appears reasonable. Subsequently, systemic therapy such as lenvatinib plus pembrolizumab can be initiated. Remarkably, after 14 months, there was no evidence of recurrence in the brain or other organs, representing an extremely rare clinical course. This case suggests that the combination of radiation therapy and LP therapy may provide effective disease control in cases of brain metastasis from endometrial cancer, although further studies are needed to validate this approach.

Many cytotoxic agents fail to cross the blood–brain barrier and have limited therapeutic efficacy in uterine cancer with brain metastases [7, 9]. When systemic disease is controlled, surgical resection and/or cranial radiotherapy is considered an appropriate option in cases of solitary brain metastases, small brain metastases less than 2 cm, no lung disease, and no extracranial lesions, as they have good outcomes [10]. However, in the present case, we judged that complete resection by surgical treatment was not possible due to the presence of multiple brain metastases and a large brain metastasis measuring more than 2 cm, and we opted for systemic chemotherapy. In many carcinomas, systemic chemotherapy is shifting from conventional cytotoxic chemotherapy to molecular targeted therapy. In study 309/KEYNOTE775 of advanced endometrial cancer, the objective response rate in the chemotherapy group was 15.1% in patients with pMMR and 12.0% in those with dMMR, whereas the combination of lenvatinib and pembrolizumab achieved ORRs of 30.3% and 40.0%, respectively, with a significantly higher response observed in the dMMR subgroup [11]. Furthermore, in the Japanese population of this clinical trial, the response rate was 36.5%, with a response rate of 62.5%, particularly for dMMR patients [12]. However, responses in patients with brain metastases in uterine cancer are unknown, as brain metastases were excluded from this clinical trial. In cases of brain metastases such as advanced renal cell carcinoma, melanoma, and non‐small cell carcinoma, the combination of lenvatinib and pembrolizumab has been reported to contribute to tumor shrinkage and prolonged survival. Although there are no reports specifically investigating the anti‐PD1 activity of immune checkpoint inhibitors in the brain, it has been demonstrated that lenvatinib inhibits tumor growth via inhibition of angiogenesis in a model mimicking brain metastasis of undifferentiated thyroid cancer [13]. This demonstrates the ability of lenvatinib to cross the blood–brain barrier. In conclusion, the combination of lenvatinib and pembrolizumab in brain metastases of endometrial cancer suggests that at least lenvatinib may have crossed the blood–brain barrier and may have been involved in the control of metastatic brain tumors.

Adverse events associated with the use of lenvatinib and pembrolizumab in patients with metastatic brain tumors include a risk of cerebral hemorrhage. In particular, lenvatinib should be administered with caution in patients with brain metastases as it is a TKI, has vasoinhibitory effects, and is associated with a risk of hemorrhage and vascular destruction due to local tumor destruction. Therefore, patients with brain metastases were exclusion criteria in Study 309/KEYNOTE775 [11]. There are few studies describing the use of TKIs for endometrial cancer brain metastases, but there is a review of reports on metastatic brain lesion hemorrhage in renal and liver cancer, where TKI use is indicated. Bleeding rates in patients with hepatocellular carcinoma who were started on TKIs after surgery or radiotherapy have been reported to be similar to those in patients who did not receive TKIs [14]. It has also been found that lenvatinib is not associated with an increased incidence of bleeding in all carcinomas for which TKIs are indicated [15]. Based on the above, the use of TKIs in patients with brain metastases appears to be a safe strategy.

Since this is a single case report, there are inherent limitations in generalizing the findings; therefore, further accumulation of similar cases and prospective studies is warranted to validate these observations.

In the present study, immune checkpoint inhibitors and lenvatinib were shown to be safe and effective treatments for cases of endometrial cancer with brain metastases. As there are few reports on the use of TKIs for brain metastases of endometrial cancer, we hope that the accumulation of additional cases will help clarify the efficacy and risks of TKI therapy in this setting. Since this is a single case report, the findings should be interpreted with caution, and further accumulation of similar cases as well as prospective studies is warranted to validate these observations.

## Author Contributions

**Ayaka Fujioka:** writing – original draft. **Akimasa Takahashi:** conceptualization, writing – review and editing, investigation, writing – original draft. **Tsukuru Amano:** writing – review and editing. **Yuji Tanaka:** writing – review and editing. **Yutaka Yoneoka:** writing – review and editing. **Shunichiro Tsuji:** supervision, writing – review and editing.

## Disclosure

An earlier version of this article was presented at the 78th Annual Congress of the Japan Society of Obstetrics and Gynecology, which was held in Okayama, Japan, on 23–25 May 2025.

## Ethics Statement

The authors have nothing to report.

## Consent

Written informed consent was obtained from the patient to publish data and images.

## Conflicts of Interest

The authors declare no conflicts of interest.

## Data Availability

Data sharing not applicable to this article as no datasets were generated or analysed during the current study.
